# Chronic Coccydynia and Coccygectomy: A Case Report and Review of the Literature

**DOI:** 10.7759/cureus.71400

**Published:** 2024-10-13

**Authors:** Abdullah Algain, Moyassar Karami, Abdulaziz Kinsara, Mahmood A Qoqandi, Hani Alsulimany

**Affiliations:** 1 Orthopedics and Spine Surgery, King Abdulaziz Medical City-Jeddah, King Abdullah International Medical Research Center, King Saud Bin Abdulaziz University for Health Sciences College of Medicine, Jeddah, SAU; 2 Orthopedics and Spine Surgery, King Abdulaziz Medical City-Jeddah, King Abdullah International Medical Research Center, Jeddah, SAU; 3 Orthopedics and Spine Surgery, Ministry of Health Jeddah, Jeddah, SAU

**Keywords:** coccydynia, coccygeal pain, coccygectomy, sacrococcygeal region pain, terminal segment of the spine

## Abstract

Coccydynia is persistent pain in the sacrococcygeal region caused by pressure on the coccyx lasting more than three months. It was treated conservatively and may mandate surgical management in the form of coccygectomy if the patient fails conservative treatment. Coccygectomy had been abandoned for a long time due to high complications. Recently, new techniques have been developed to overcome these complications. In this article, we present a case of a 41-year-old male with chronic coccydynia who did not respond to non-operative measures, and surgical management involving coccygectomy was performed with specific intraoperative and postoperative protocols. Further studies are needed to attract more researchers to investigate coccygectomy further.

## Introduction

Coccydynia refers to pain in the sacrococcygeal region resulting from pressure on the coccyx, also known as the tailbone. Coccydynia is mainly idiopathic and develops from unknown causes. However, direct injuries to the sacrococcygeal region such as falling from a height and challenging vaginal delivery are known to be the cause in several cases [1]. Coccydynia is five times more common in females than males [2]. Obesity and advancing age are other contributing factors [3]. The presentation is characterized by tenderness at the terminal segment of the spine, which is usually exacerbated by prolonged sitting. The pain varies in severity and may radiate superiorly to the lumbosacral region or laterally to the buttock region [4]. The entity can mimic other conditions, such as pilonidal cysts and sacroiliitis, which may contribute to delayed diagnosis or sometimes misdiagnosis [5]. Diagnosis is primarily clinical depending on history and physical examination. Various imaging modalities aid in the diagnostic process, contributing to a precise and informed clinical decision. Plain radiographs, for example, are used to rule out other possible conditions [5]. However, magnetic resonance imaging (MRI) is preferred due to its ability to detect the specific site of irritation and provide better visualization of the coccyx [6]. There is no definite protocol for managing coccydynia since it relies on several factors, making the treatment somewhat complicated [7]. Nevertheless, warm baths, non-steroidal anti-inflammatory drugs (NSAIDs), and physiotherapy tend to be effective for acute and mild coccydynia. Further interventions like regional steroid injections and coccygeal manipulation are used in certain cases [8].

Chronic refractory coccydynia, defined as persistent pain for more than three months, may mandate surgical management in the form of coccygectomy [8]. Refractory coccydynia is associated with extreme pain in the sacrococcygeal region, which may interfere with daily activities and cause physical disabilities. However, coccygectomy has shown significant improvement in outcomes regarding the alleviation of this pain. A single institution series study assessed preoperative and postoperative pain scores using the visual analog scale (VAS). The preoperative pain intensity, as measured by the VAS score, had a mean of 6.97 (SD = 1.99). Postoperatively, the pain intensity decreased significantly, with a mean of 5.80 (SD = 2.11). This indicates a reduction in pain following the surgery, as evidenced by the lower postoperative VAS score compared to the preoperative score [9].

The standard technique for coccygectomy typically involves making a vertical midline incision near the coccyx, exposing the bone, and then carefully removing part or all of the coccyx and its ligamentous attachments. However, the technique may vary slightly depending on the surgeon's preference and the patient's condition. In a retrospective study [1], a comparison was done between two different techniques, removal of the coccyx with and without periosteal resection, to observe the efficacy of both methods. Patients were subdivided into two groups. The first group underwent coccygectomy that involved coccygeal attachments, including cutting of anococcygeal ligament, coccygeus, and iliococcygeus muscles, in addition to resection of the periosteum. The second group underwent preserved ligamentous and muscular attachments along with the periosteum. The study showed that both surgical techniques resulted in a high success rate and statistically similar clinical outcomes regarding pain assessment and subjective patient rating. Although the procedure has a high success rate, some complications may occur such as wound infection, bleeding, and nerve injury [7]. One way to minimize complications and reduce the risk of developing wound dehiscence and infection is using the Z-plasty technique for wound closure. This technique involves creating triangular flaps of tissue and repositioning them to change the direction of scars, reduce tension, and improve healing. While the conventional approach, which involves a vertical midline incision, is associated with infection rates ranging from 14% to 30%, applying the Z-plasty technique resulted in 100% absence of infection or any wound-related problem [7]. The prognosis of the condition can be influenced by different factors. Studies have shown that patients who developed coccydynia from physical trauma and patients with a BMI less than 30 responded better to treatment modalities such as local steroid injections and coccygectomy [3].

## Case presentation

**Figure 1:**
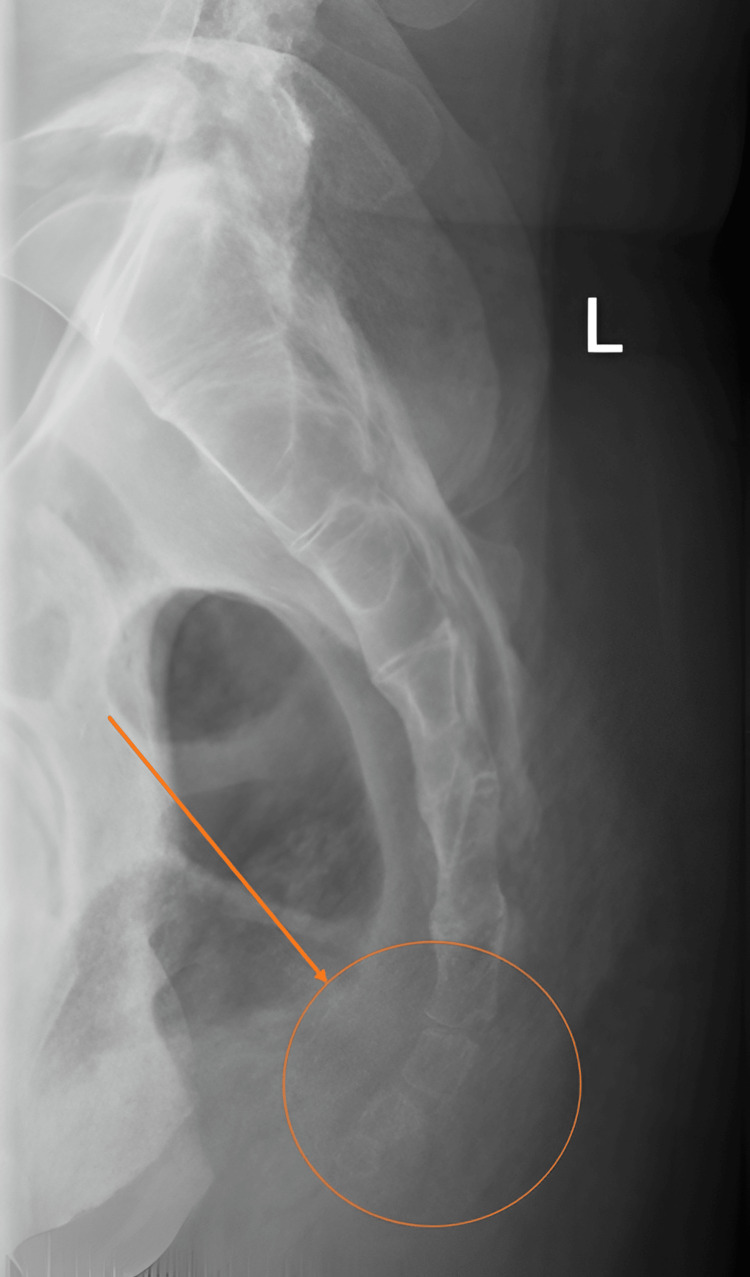
Lateral radiograph of the sacrum and coccyx showing the normal five sacral and four coccygeal segments

**Figure 2:**
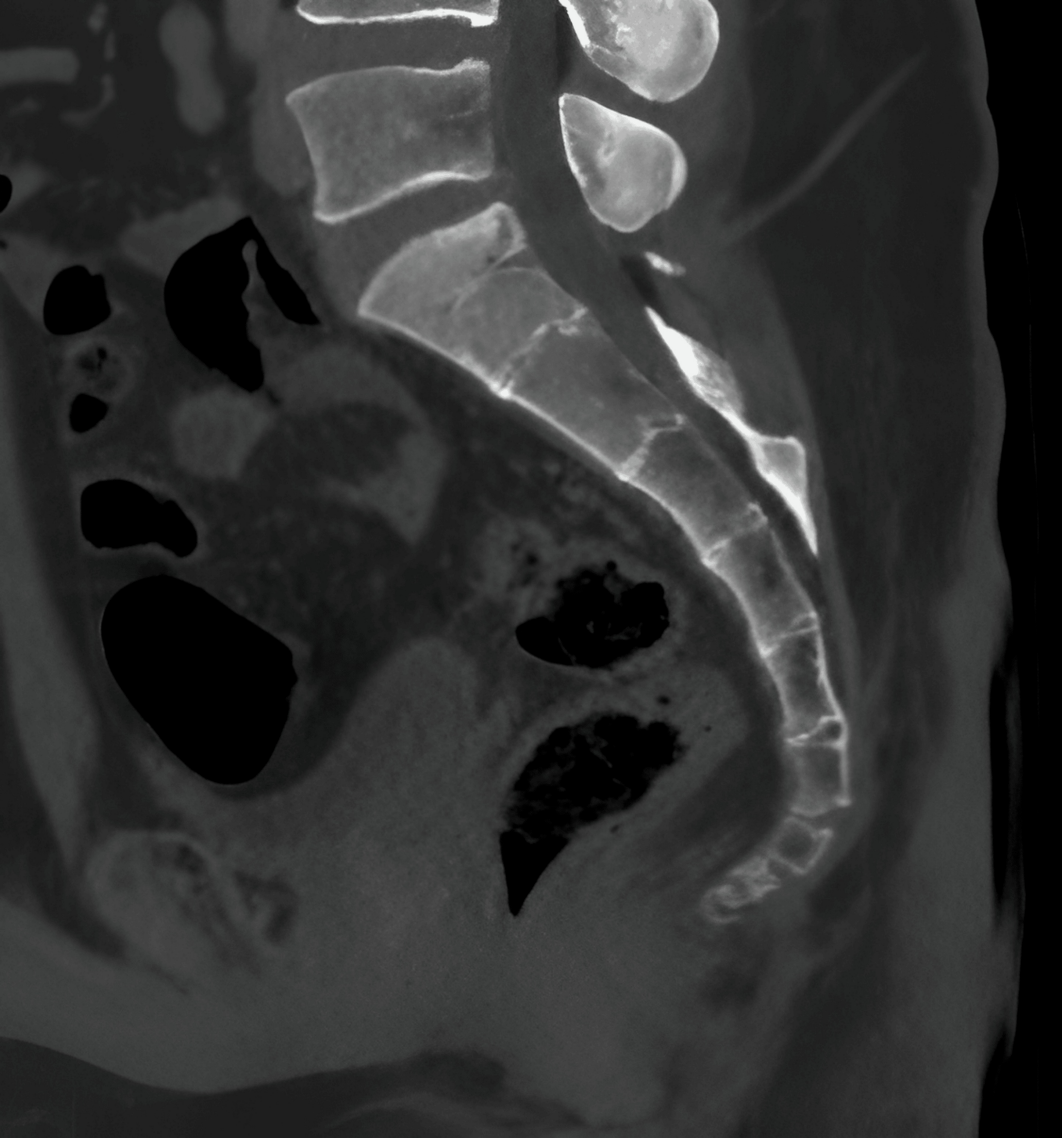
Sagittal CT image of the sacrum and coccyx showing the normal architecture of the spine

**Figure 3:**
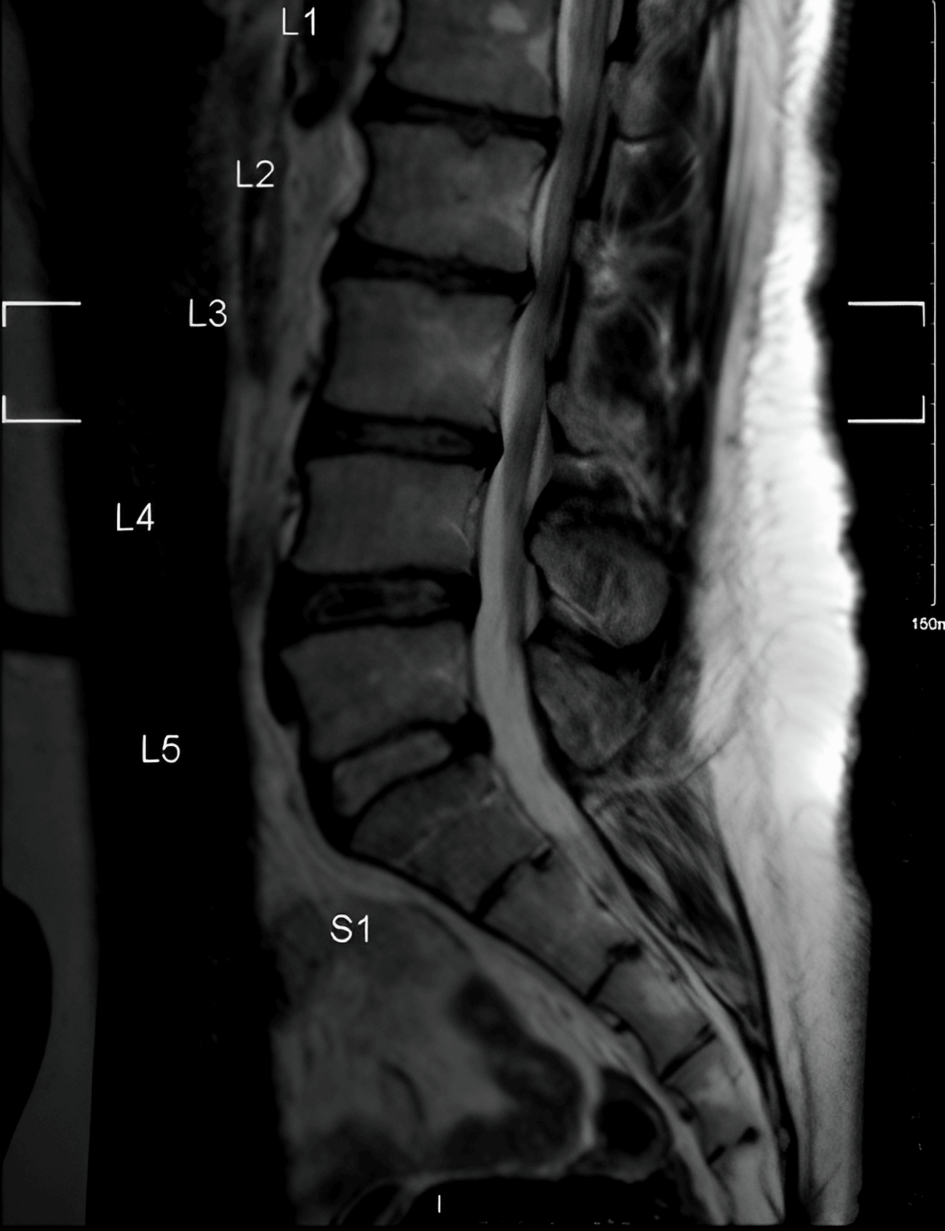
Midline sagittal T2-MRI image showing a normal lower spine segment with mild degenerative changes

A 41-year-old male presented in 2016 with chronic coccydynia for 10 years. This coccygeal pain radiated to his gluteal area and was typically aggravated by prolonged sitting. The pain varied in severity over time until it became so intense that it reached a pain score of 9/10, causing him an inability to sit. The patient did not have any medical illnesses, with a history of previous gastric sleeve and anal fistula repair. Different imaging and screening modalities were used to assess the patient’s condition, including sacrococcygeal X-ray, pelvis CT scan, and lumbar MRI (Figures 1-3). The patient underwent and failed all non-operative modalities, including NSAIDs, donut-cushioned pillows, physiotherapy, and steroid injections. Therefore, a coccygectomy was offered, and the patient agreed.

Operative details

Before the procedure, the patient was commenced on a soft diet for 48 hours. He received two doses of Fleet Enema. The first dose was administered in the evening time one day before the surgery. The second was given early morning on the same day of the surgery.

Cefazolin 2 g and metronidazole 100 mg were used as prophylactic antibiotics before skin incision. The patient was positioned prone on a standard Jackson table. Gentle traction of the skin and the gluteal area was done using plaster to further expose the midline crease. Under a C-arm, a vertical skin mark was made over the coccygeal area. The perianal area was covered with iodine-soaked gauze distal to our intended incision. Prepping and draping were done in the usual sterile fashion.

A vertical incision was made over the coccygeal area. Dissection of the soft tissue was carried down to the bone using a monopolar. Subperiosteal dissection was carefully continued from the midline posteriorly down to the lateral side bilaterally. Using a blade, the sacrococcygeal disk was removed. Then using towel clips to direct the coccyx, dissection was carried from the posterolateral side into the ventral side while maintaining subperiosteal dissection. Hemostasis was controlled using monopolar and coagulation. Then the coccygectomy was completed, and no rectal injury was encountered. Anteroposterior and lateral radiographs were taken to confirm the complete removal of the bone. The distal edge of the sacral bone was rasped, and bone wax was applied. Thorough irrigation and local vancomycin were applied. Before closure, the traction was released. Closure was done in multiple layers to eliminate the dead space area. Deep fascial closure was done using 2-Vicryl where the periosteum was sutured as well. The superficial fascia was closed using 0-Vicry and the subcutaneous fascia using 2-0 Vicryl. The skin was closed using Monocryl and reinforced by an interrupted simple non-absorbable suture. Dermabond glue was also applied to the wound along with Steri-Strips. Two layers of dressing were applied. The first layer was applied to the edge of the wound, while the second was proximal to the perianal area, with gauze applied between the two layers.

Postoperative management

**Figure 4:**
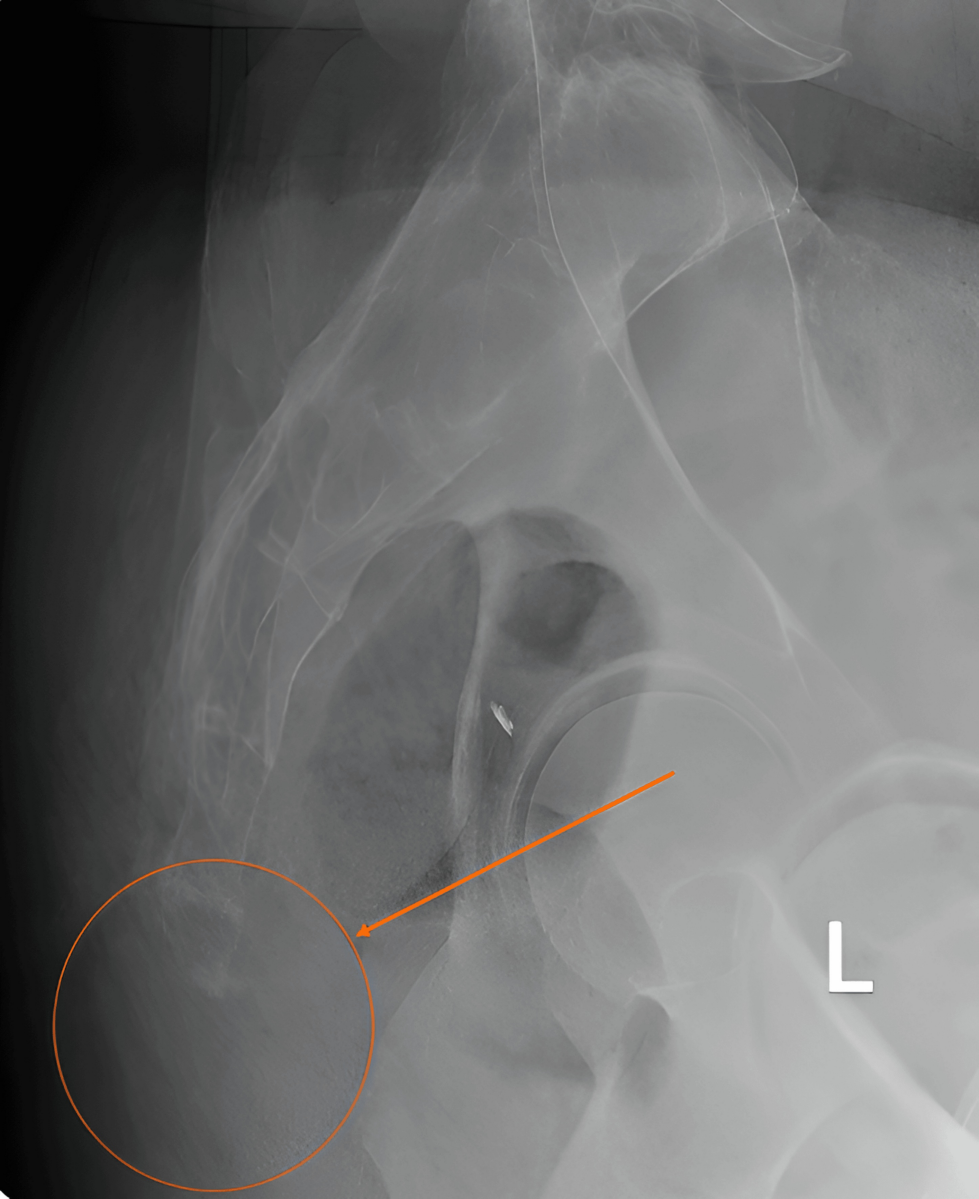
Postoperative lateral radiograph of the sacral-coccygeal region showing the five sacral bones with resected coccygeal segment (orange arrow)

The patient was discharged the day after the surgery. He was advised to avoid sitting and follow a high-liquid and fiber diet with laxatives to prevent constipation. The wound was clean in his first postoperative outpatient visit. By the second visit at two weeks, the wound showed minor distal gap healing, and sutures were removed. The patient showed mild wound sloughing on his third visit. Four weeks post-surgery, the wound was healed completely with a pain score of 3/10 compared to baseline. Furthermore, the patient was followed for more than 12 months reporting a significant reduction in his pain and no limitation of activity (Figure 4).

## Discussion

Coccydynia is a painful condition that can present acutely or chronically. The pain commonly affects the sacrococcygeal area. There are multiple causes including the idiopathic type [1]. It affects females more than males [2]. Coccydynia is not an uncommon condition. Management of coccydynia poses a clinical challenge as there is no standard treatment protocol [7]. Investigating patients with suspected coccydynia includes erect and sitting sacral radiographs in addition to MRI. Various treatment options exist from oral anti-inflammatories, local injections, and surgical treatment. Coccygectomy has a success rate of up to 90%; however, complications can reach up to 30% [7]. In this article, we present our experience in performing a coccygectomy on a 41-year-old male who failed to respond to non-operative modalities. In 1936, Key published his technique for performing coccygectomy. Years later, coccygectomy fell out of favor due to a high complication rate including infection. Different protocols and alterations have been described to avoid these complications. In our case, the preoperative and postoperative protocols were thoroughly discussed with the patient. There are three main considerations for performing proper coccygectomy. First, preoperative bowel preparation. Second, intraoperative careful dissection to avoid rectal injuries and tight closure. Lastly, patient compliance for a postoperative period, taking into consideration a semi-solid diet for one week and keeping the wound dry [10].

Avoiding constipation immediately postoperatively is paramount to help reduce strain on the surgical wound. This can be achieved by a combination of preoperative soft diet initiation, bowel preparation, and postoperative laxatives. The preoperative cleansing program protocol starts one week before surgery and involves a transition from a semi-solid diet to a liquid-only diet during the final two days before the surgery [10]. We commenced our patient on a soft diet for 48 hours before the surgical intervention. Moreover, he received two doses of Fleet Enema within 24 hours of the surgical intervention. A similar protocol was also reported in the literature [1].

There are multiple described modifications for performing coccygectomy. These variations are based on the surgeon’s preference. Kulkarni et al. [7] reported no wound infections using the Z-plasty technique. In our case, we performed a midline vertical skin incision technique. Another consideration is the resection versus preservation of the periosteum and surrounding ligaments [1]. His group compared both techniques and found no significant difference. In our experience, closure of the soft tissue including the periosteum was performed tightly to reduce the dead space. We emphasize releasing the traction of the soft tissues to allow tight closure in a layer-by-layer fashion. In addition, careful subperiosteal dissection to avoid rectal injuries is paramount. Skin closure protocol varies; however, we performed continuous skin closure using Monocryl, reinforced by simple interrupted Prolene, with Dermabond Glue used to seal the surface for any contamination.

Success in performing coccygectomy extends to the postoperative period. Proper wound care and avoidance of strain should not be taken lightly. Sitting position should be avoided to reduce strain on the wound. This will reduce the pressure on the surgical site and create a suitable environment for healing. The latter protocol was also studied and supported in the literature as described by Kwon et al. [4]. The postoperative diet protocol consists of a high-fiber and liquid diet in addition to laxatives to avoid constipation. The patient was discharged one day after the surgical intervention with home instructions. His postoperative course went uneventfully until the two-week visit. He developed wound dehiscence distally with no signs of infection. Local wound care protocols were applied, and the wound was healed within four weeks of the operative intervention. At the four-week visit, he reported a decrease in pain with a score of 3/10. He completed more than one year of postoperative follow-up, his pain level remained at 3/10, and he has no limitations in his daily activities.

## Conclusions

Coccygectomy is an old procedure that was abandoned due to its high complication rate despite successful pain control. Emerging evidence suggests lower complications and successful outcomes using newly developed protocols. We report our experience in performing coccygectomy, which resulted in good pain control, functional outcomes, and fully recovery from minor complications.
